# The effects of neuregulin-1β on intrafusal muscle fiber formation in neuromuscular coculture of dorsal root ganglion explants and skeletal muscle cells

**DOI:** 10.1186/s13395-018-0175-9

**Published:** 2018-09-15

**Authors:** Yuan Qiao, Menglin Cong, Jianmin Li, Hao Li, Zhenzhong Li

**Affiliations:** 1 Department of Anatomy, Shandong University School of Basic Medical Sciences, 44 Wenhua Xi Road, Jinan, 250012 Shandong Province China; 2 Department of Orthopaedics, Shandong University Qilu Hospital, Jinan, 250012 China

**Keywords:** Neuregulin-1, Intrafusal muscle fiber, Skeletal muscle, Neurite outgrowth, Growth-associated protein 43, Tyrosine kinase receptor C

## Abstract

**Background:**

The formation of intrafusal muscle (IM) fibers and their contact with afferent proprioceptive axons is critical for construction, function, and maintenance of the stretch reflex. Many factors affect the formation of IM fibers. Finding new factors and mechanisms of IM fiber formation is essential for the reconstruction of stretch reflex arc after injury.

**Methods:**

We established a coculture system of organotypic dorsal root ganglion (DRG) explants and dissociated skeletal muscle (SKM) cells. The formation of IM fibers was observed in this coculture system after neuregulin-1β (NRG-1β) incubation.

**Results:**

We found that NRG-1β promoted outgrowth of neurites and migration of neurons from the organotypic DRG explants and that this correlated with an induction of growth-associated protein 43 (GAP-43) expression. NRG-1β also increased the amount of nuclear bag fibers and nuclear chain fibers by elevating the proportion of tyrosine kinase receptor C (TrkC) phenotypic DRG neurons. In addition, we found that the effects of NRG-1β could be blocked by inhibiting ERK1/2, PI3K/Akt, and JAK2/STAT3 signaling pathways.

**Conclusion:**

These data imply that NRG-1β promoted neurite outgrowth and neuronal migration from the organotypic DRG explants and that this correlated with an induction of GAP-43 expression. The modulating effects of NRG-1β on TrkC DRG neuronal phenotype may link to promote IM fiber formation. The effects produced by NRG-1β in this neuromuscular coculture system provide new data for the therapeutic potential on IM fiber formation after muscle injury.

**Electronic supplementary material:**

The online version of this article (10.1186/s13395-018-0175-9) contains supplementary material, which is available to authorized users.

## Background

New recognized multi-sources of neuregulin-1 (NRG-1) from both neurons and skeletal muscle (SKM) attract scientists to explore its functions on multi-targets or multi-organs [1]. NRG-1 signaling is critical for the normal development of the nervous system [2] and neuro-repair after injury [3, 4]. It has been shown that NRG-1 signaling has the capability on improving muscle contraction during myogenesis in vitro [5] and on maturation of the muscle spindle for maintaining motor coordination in vivo [6]. Interestingly, it has been shown that NRG-1 through Gab1 has regulatory effects on myelination of the peripheral nerve [7], postnatal development of neuromuscular junction (NMJ) [8], and development of the extrafusal muscle (EM) fibers and intrafusal muscle (IM) fibers [9]. Endogenous NRG-1 released from sensory nerve endings by targeting its ErbB receptors in post-junctional muscle cells is involved in the formation of muscle spindles [10] as well as NMJ [11]. Interestingly, NRG-1 plays an important role on NMJ development in fine-tuning pre-, post-, and perisynaptic specialization [12]. Furthermore, NRG-1 signaling is essential for fusimotor innervation homeostasis, IM fiber differentiation, and spindle morphogenesis and function [13]. NRG-1 by inducing phosphorylation of ErbB2 receptors has a specific driving effect on myotubes in serum-free culture to the nuclear bag phenotype [14]. However, the effects of exogenous NRG-1 on the development of IM fibers in the presence of sensory neurons are still unknown.

The development and maturation of IM fibers prior to EM fibers are needed for the proprioceptive demands and early reflex control in developing motor skills [15]. Different from numerous EM fibers, IM fibers within muscle spindles are specialized muscle fibers which retain features characteristic of immaturity [16] and are included nuclear bag fibers and nuclear chain fibers. In muscle spindles in vivo, the nuclear bag fibers are fusiform in shape with gathered nuclei inside in the central part of the muscle fiber, and the nuclear chain fibers are thinner than the nuclear bag fibers with linear assembled nuclei inside the muscle fiber. In culture, the shape of the nuclear bag and nuclear chain fibers is different. The shape of bag fibers is fusiform with gathered nuclei inside the muscle fiber. Chain fiber is thin cylindrical in shape with linear assembled nuclei inside the muscle fiber. The IM fibers in culture are dispersed in distribution which are different from that they are gathered within muscle spindles in vivo. The mechanical sensory receptor muscle spindle, which is sensitive to muscle length alteration and related to movement and posture control, is composed of IM fibers and the sensory nerve endings around them [17, 18]. Muscle spindle inputs were also involved in regulating isometric muscle contraction [19]. The sensory nerve endings along with IM fibers in the central region are the end part of the peripheral processes of the pseudounipolar dorsal root ganglion (DRG) neurons [20–24]. Activation of tyrosine kinase receptor C (TrkC) not only mediates health, recovery, and function of motor neurons [25–27]; TrkC-positive neurons also represent the subtype of proprioceptors in DRG [28–32]. TrkC proprioceptive DRG neurons with their peripheral axons around IM fibers transmit information from muscle spindles to the spinal cord through central processes [33, 34]. The development or maturation of TrkC phenotypic DRG neurons is dependent on the simultaneously developing target SKM cells [35]. Proprioceptive DRG sensory neurons degenerate with dysregulated TrkC signaling prior to IM fiber atrophy during aging [36], suggesting the close relationship between IM fibers and TrkC phenotypic DRG neurons.

The initiation of growth-associated protein 43 (GAP-43) is closely correlated to the promotion of nerve regeneration and repair in injured mature neurons [37–40] and is involved in the orientation of cell division in dividing neural progenitor cells for neurogenesis [41]. The evaluation of GAP-43 expression could be a useful quality indicator for assessment of nerve regeneration [42–46]. GAP-43 also has a pivotal role in regulating axon functional plasticity and recovery as well as structural plasticity and reconstruction [47–50]. Reduced GAP-43 indicates neurite degeneration of DRG neurons [51]. GAP-43 expression in nerve fibers is related to the activation of Akt/mTOR signaling after spared nerve injury [52]. GAP-43 expression-related neurite elongation of DRG neurons is through the activation of Etv4 and Etv5 transcription factors [53]. Interestingly, expression of GAP-43 and TrkC was upregulated simultaneously in DRG during proprioception recovery [54]. In the present study, GAP-43 expression would be used as an indicator of DRG neuronal growth status in the presence of target SKM cells and exogenous NRG-1β in vitro.

The amount of muscle spindles with their normal neuronal innervation is crucial for efficacy of proprioceptive regulation [55]. Formation and specific differentiation of muscle spindles involve mutual interactions of developing IM fibers and afferent axons that directly innervate them [22]. Interestingly, the innervation patterns of nuclear bag fibers differ from those of nuclear chain fibers [56]. During development and regeneration, IM fibers also express unique myosin isoforms as well as EM fibers [57]. Diabetes-induced neuronal loss may induce muscle spindle atrophy in rats [58]. In RYR1-related myopathy mice, IM fiber abnormalities prior to EM fiber alterations imply that the IM fiber is friable and is a primary pathological feature in this degenerated myopathy [59]. Activation of the critical components of NRG-1’s intracellular pathways is related to muscle spindle formation. Herndon et al. confirmed that NRG-1 is required for the activation of ERK1/2 signaling pathway in response to induce the transcription factors in IM fibers of the muscle spindles [60]. As a highly plastic tissue, activation of the Akt intracellular signaling pathway is a crucial step in regulating the growth of muscle fibers [61]. Lai et al. found that the concentrated JAK2/STAT3 proteins related to NMJ formation in adult muscle are confirmed by using the cultured C2C12 myotubes with the JAK2 inhibitor AG490 to inhibit the phosphorylation of STAT3 [62]. However, how and to what extent NRG-1β influences the formation of IM fibers in the presence of both DRG neurons and developing muscle cells should be further evaluated.

In vitro phenotypic model is beneficial for illustrating functional neuromuscular reflex arc development [63]. In this study, it is hypothesized that NRG-1β could induce neurite outgrowth of DRG neurons and the formation of IM fibers in a coculture system of DRG explants and dissociated muscle cells. The activation of downstream signaling pathways ERK1/2, PI3K/Akt, and JAK2/STAT3 may be related to the effects of NRG-1β. The TrkC neuronal phenotype may have specific functions on the formation of IM fibers. The specific effects of NRG-1β and its downstream signaling on the formation of IM fibers and its involvement in TrkC phenotypic DRG neurons will be studied in this experiment. ERK1/2 inhibitor PD98059, PI3K inhibitor LY294002, and JAK2 inhibitor AG490 were used as the effective downstream signaling inhibitors in NRG-1 experiments, and these inhibitors would be utilized in our present study [62, 64]. Although the addition of NRG-1β to induce the formation of IM fiber had been studied before, the results will provide novel data for the therapeutic potential on IM fiber formation after muscle injury with NRG-1β administration.

## Methods

### Coculture of organotypic DRG explants and dissociated SKM cells

This coculture means organotypic DRG tissue explants and dissociated SKM cells would grow in the same well of the clusters and form neuromuscular junction (NMJ)-like structure in vitro. The procedure of the coculture was performed similarly as our previous study [65]. The DRG explants and dissociated SKM cells were prepared separately at different time points.

SKM cell culture preparations utilize newborn rats taken from the breeding colony of Wistar rats maintained in the Experimental Animal Center at Shandong University of China. SKM cell cultures were prepared 2 days prior to DRG preparation. In brief, muscles from the limbs of neonatal rats were collected and cut into fragments approximately 0.5 mm in diameter. After digestion with 0.25% trypsin (Sigma) in D-Hanks solution at 37 °C for 40 min, the cell suspension was filtered through 200 mesh, centrifuged, and triturated in growth media supplemented with 10% fetal bovine serum (Gibco, Origin, Australia). Isolated SKM cells were plated at a density of 2 × 10^5^ cells/mL in 24-well clusters (Costar, Corning, NY) which would contain a coverslip precoated with poly-l-lysine (0.1 mg/mL) in each well. The 24-well clusters with isolated SKM cells were then placed in an incubator with proper culture environment at 37 °C and 5% CO_2_.

The DRG culture preparations utilized rat embryos at embryonic day 15 (E15). Under aseptic conditions, the bilateral dorsal root ganglia (DRGs) were removed from each embryo and placed in culture media in the half of petri dishes. Each DRG explant was plated at the bottom of each well of 24-well clusters (Costar, Corning, NY) which contained single-layered SKM cells.

**Fig. 1:**
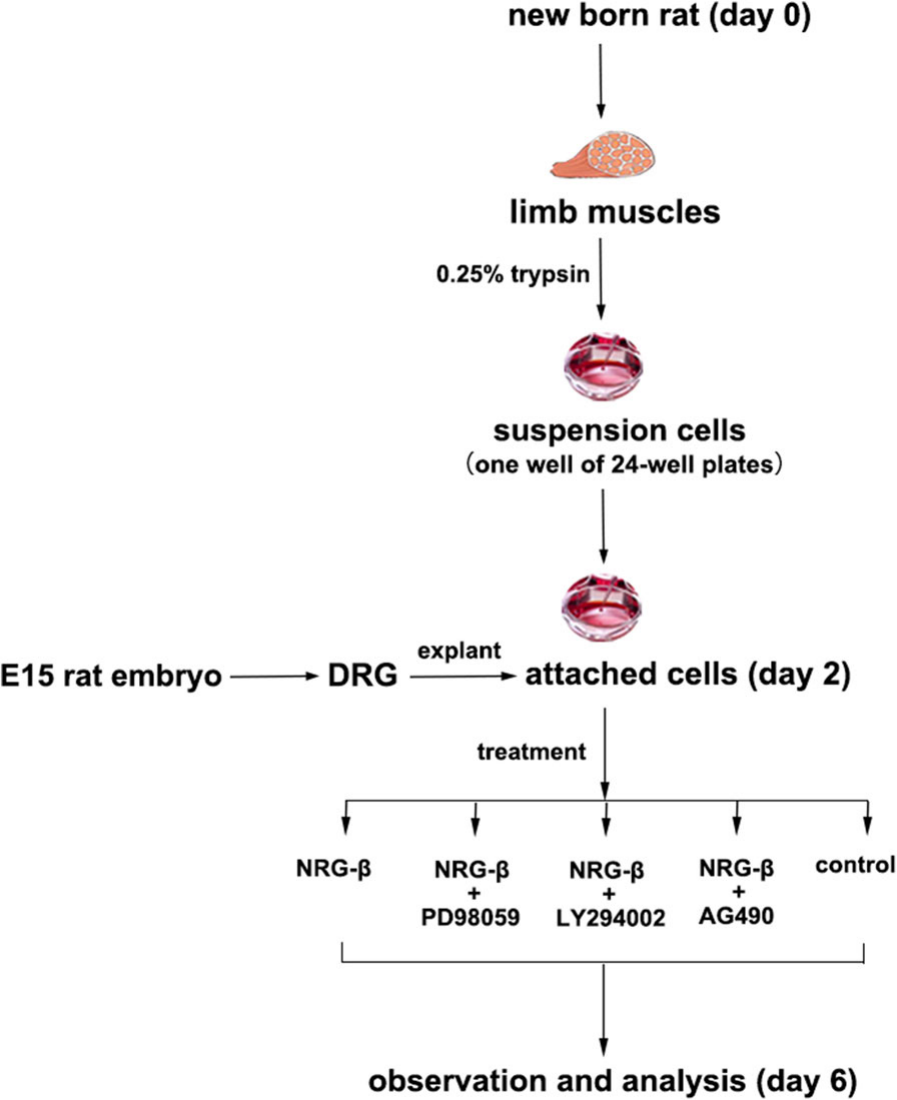
The schematic diagram of protocols for neuromuscular coculture preparations and treatment timeline

The neuromuscular coculture was prepared as follows: Each newly prepared DRG explant was plated into a well with 2-day-old SKM cell culture after confluent myoblast fusion has happened. The neuromuscular cocultures of organotypic DRG tissue explants and dissociated SKM cells were allowed to grow for an additional 4 days with media change every 2 days. The protocols for neuromuscular coculture preparations and treatment timeline were shown in Fig. 1.

### Treatment with different agents after coculture establishment

The established cocultures were randomly divided into the following groups with the corresponding treatment: (1) NRG-1β: The coculture was treated with NRG-1β (20 nmol/L, Peprotech, Rocky Hill, NJ). (2) NRG-1β + PD98059: ERK1/2 inhibitor PD98059 (10 μmol/L, Cell Signaling Technology) was applied 30 min prior to NRG-1β (20 nmol/L) treatment. (3) NRG-1β + LY294002: PI3K inhibitor LY294002 (10 μmol/L, Invitrogen) was applied 30 min prior to NRG-1β (20 nmol/L) treatment. (4) NRG-1β + AG490: JAK2 inhibitor AG490 (10 μmol/L, Invitrogen) was applied 30 min prior to NRG-1β (20 nmol/L) treatment. (5) Control group: The cells were cultured continuously in media. All aforementioned incubation conditions were maintained in 37 °C and 5% CO_2_ environment with the corresponding agents for 4 days with media change every 2 days.

### Living cell observation

Living cell observation was for counting the number of neurite bundles sent by organotypic DRG tissue explants. The length of neurite bundles included in this counting is longer than 200 μm. Only the number of neurite bundles in the superior lateral quadrant was included for each DRG explant.

### Microscopy of fluorescence labeling

The microtubule-associated protein 2 (MAP2) was used to stain the migrating DRG neurons from the explants. Double fluorescence staining with neurofilament 200 (NF-200) for neuron and α-actin for SKM cell was used for determining the formation of nuclear bag or nuclear chain fibers. Triple fluorescence staining experiment was carried out with NF-200 for neuron, TrkC for specific TrkC phenotype, and α-actin for SKM cell. The fluorescent staining procedures were done as our previous study [65]. The antibodies used in this experiment were shown in Table 1. The morphology of the annulospiral sensory nerve endings after NRG-1β treatment was observed under maximum optical resolution by analyzing projection from sensory neurons in contact with SKM cells in the DAPI-labeled nuclei SKM cell area. The nuclear bag or nuclear chain fibers were counted separately in each sample. This coculture system represents the formation of synaptic specializations, particularly muscle spindles, but the connective tissue capsule structures surrounding IM fibers could not be visualized. Therefore, the number of nuclear bag or nuclear chain fibers was counted, respectively, for comparison. Moreover, triple fluorescence labeling of NF-200, TrkC, and α-actin was performed to observe the in situ expression of TrkC-positive neurons and the proportion of TrkC-positive neurons with their neurites contacted with muscle fibers.

**Table 1:** The primary and secondary antibodies for fluorescence labeling

Category	Antibodies	Concentration	Source
Primary	Chicken polyclonal anti-MAP2	1:1000	Abcam, Cambridge, MA
Primary	Chicken polyclonal anti-NF-200	1:1000	Abcam, Cambridge, MA
Primary	Mouse polyclonal anti-α-actin	1:50	Abcam, Cambridge, MA
Primary	Rabbit anti-TrkC polyclonal IgG	1:500	Abcam, Cambridge, MA
Secondary	Goat anti-mouse IgG conjugated to DyLight 350	1:400	ImmunoReagents, Chicago, IL
Secondary	Goat anti-chicken IgG conjugated to DyLight 488	1:400	ImmunoReagents, Chicago, IL
Secondary	Goat anti-rabbit conjugated to Cy3	1:400	Abbkine, Redlands, CA
Secondary	Goat anti-mouse IgG conjugated to TRITC	1:200	ZSGB-BIO, Beijing, China

### Quantification of neurons and IM fibers after fluorescence labeling

Neuronal migration from organotypic DRG tissue explants was defined as one visual field at the superior lateral quadrant adjacent to the lateral border of the explants in each sample. The counting example was shown in “Additional file 1: Figure S1”.

The morphology of the annulospiral sensory nerve endings after NRG-1β treatment was observed under maximum optical resolution by analyzing projection from sensory neurons in contact with SKM cells in the DAPI-labeled nuclei SKM cell area. Only nuclear bag fibers and nuclear chain fibers were taken into account in four visual fields in the upper, lower, left, and right sides of DRG explant in each sample. This coculture system of DRG neurons and SKM cells represents the formation of synaptic specializations, particularly muscle spindles, but the connective tissue capsule structures surrounding IM fibers could not be visualized. Therefore, the number of both nuclear bag fibers and nuclear chain fibers were counted, respectively, for comparison.

Moreover, triple fluorescence labeling of NF-200, TrkC, and α-actin was performed to observe the in situ expression of TrkC-positive neurons. The percentage of TrkC-positive neurons, which innervate muscle fibers, in one visual field at the superior lateral quadrant adjacent to the lateral border of the explants was counted in each sample.

### Real-time PCR for mRNA expression of GAP-43 and TrkC

After treatment with different agents for 4 days, the mRNA levels of GAP-43 and TrkC in the cocultures were measured by using real-time PCR with GAPDH mRNA as an internal control. TRIzol was used for extracting total RNA from DRG cells. Before reverse transcription, DNAse treatment was carried out to avoid DNA contamination in RT-PCR. cDNA synthesis kit (Fermentas) was used for synthesizing cDNA with the instructions from the manufacturer. The primer sequences of synthetic oligonucleotide for each detecting gene were shown in Table 2. SYBR Green dye (Fermantas) was used for real-time PCR with the instructions from the manufacturer. PCR was carried out at 50 °C for 2 min, 94 °C for 15 min, followed by 40 cycles at 94 °C for 15 s, 58 °C for 30 s, and 72 °C for 30 s. By using the 2^−ΔΔCt^ method, target gene relative transcript amount was normalized to GAPDH gene from a comparative cycle of threshold fluorescence (Ct) method.

**Table 2:** The sequences of oligonucleotide primers

Genes	Primer sequences	Length after amplification
GAP-43	5′-AAG AAG GAG GGA GAT GGC TCT-3′ (forward)	197
	5′-GAG GAC GGC GAG TTA TCA GTG-3′ (reverse)	
TrkC	5’-CCC ACT ACA ACA ATG GCA ACT A-3′ (forward)	187
	5’-CCA AAA GTG TCT TCC TCT GGT T-3′ (reverse)	
GAPDH	5’-GGC ACA GTC AAG GCT GAG AAT G-3′ (forward)	143
	5′-ATG GTG GTG AAG ACG CCA GTA-3′ (reverse)	

### Western blot assay for protein levels of GAP-43 and TrkC

After treatment with different agents for 4 days, GAP-43 and TrkC expression in the cocultures were measured by using Western blot. RIPA Lysis Buffer with protease inhibitors (Amersco) was used for homogenizing cells. The supernatant was collected after centrifuge (10,000×*g*, 20 min) for concentration measurement (BCA method, standard: BSA), followed by 10% SDS gel loading, separating with electrophoresis, and PVDF membrane transferring. The following membrane blocking and incubation with first and second antibodies were done as our previous study [65]. The antibodies used in this experiment were shown in Table 3.

**Table 3:** The primary and secondary antibodies for Western blot assay

Category	Antibodies	Concentration	Source
Primary	Rabbit anti-GAP-43 monoclonal IgG	1:100,000	Abcam, Hong Kong
Primary	Rabbit anti-TrkC polyclonal IgG	1:1500	Abcam, Cambridge, MA
Primary	Mouse anti-β-actin monoclonal IgG	1:4000	Santa Cruz Biotechnology, Santa Cruz, CA
Secondary	Goat anti-rabbit IgG-HRP	1:4000	Santa Cruz Biotechnology, Santa Cruz, CA
Secondary	Goat anti-mouse IgG-HRP	1:3000	Santa Cruz Biotechnology, Santa Cruz, CA

### Statistical analysis

Each condition was repeated three times for one sample. Five samples (*n* = 5) were used for statistical analysis for all the experiments. The mean ± SD was used for reporting the data. Statistical analysis was carried out with SPSS software (19.0). One-way ANOVA was selected for analyzing the data from this study. Sub-statistical method S-N-K test was performed with data for homogeneity of variance. Dunnett’s T3 test was performed with data for heterogeneity of variance. Significant evaluation was defined as *P* < 0.05.

## Results

### The neurite outgrowth from DRG explants

**Fig. 2:**
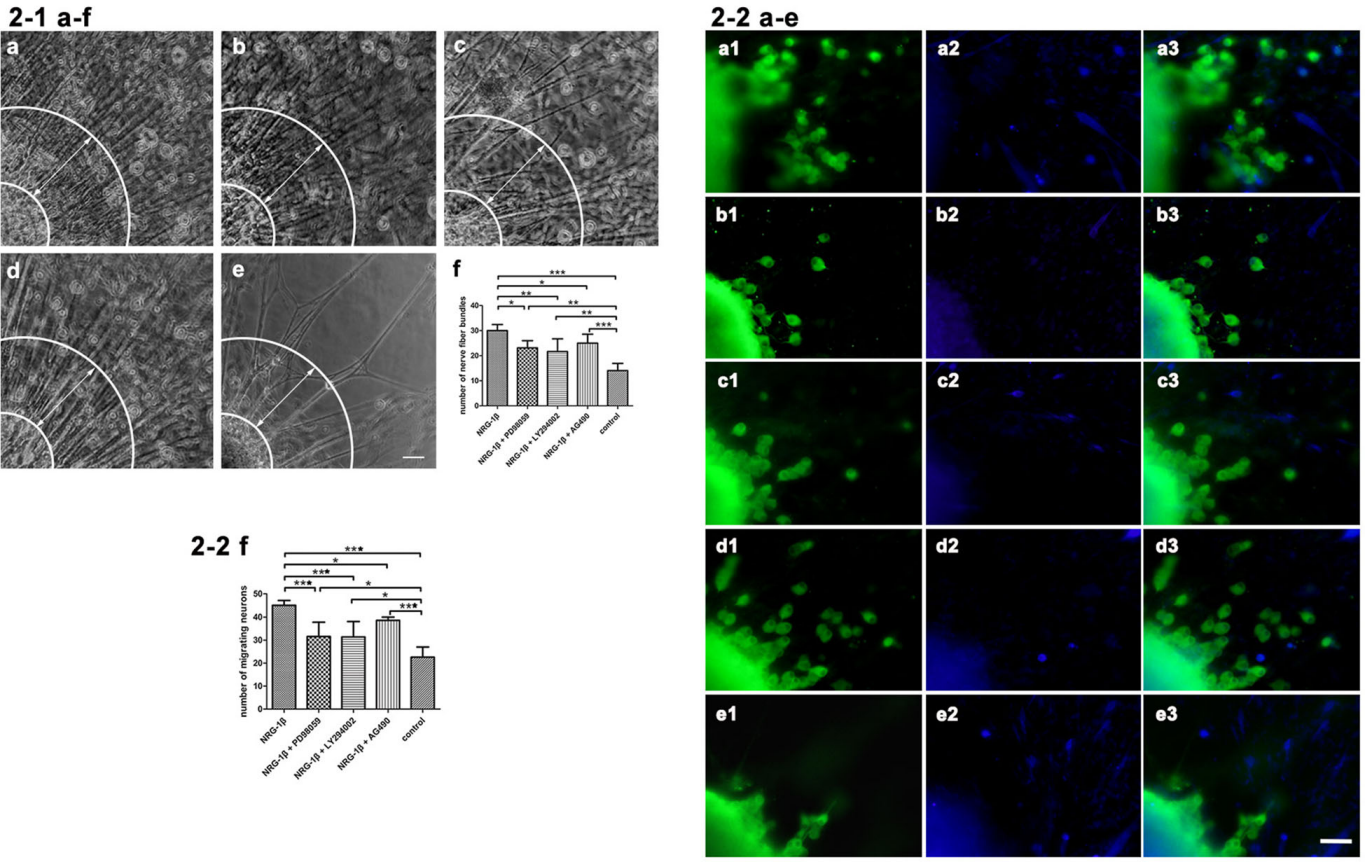
Neurite bundles and migrating neurons from organotypic DRG explants. **2-1 a**–**e** The neurite bundles (> 200 μm in length) in the superior lateral quadrant were counted in each sample. **2-1 a** NRG-1β. **2-1 b** NRG-1β + PD98059. **2-1 c** NRG-1β + LY294002. **2-1 d** NRG-1β + AG490. **2-1 e** control. Scale bar = 50 μm. **2-1 f** Quantification of the number of nerve fiber bundles. Mean ± SD, *n* = 5. *P < 0.05, **P < 0.01, ***P < 0.001. **2-2 a**–**e** The migrating neurons from organotypic DRG explants in different conditions. **2-2 a** NRG-1β. **2-2 b** NRG-1β + PD98059. **2-2 c** NRG-1β + LY294002. **2-2 d** NRG-1β + AG490. **2-2 e** control. Scale bar = 50 μm. **2-2 f** Quantification of the migrating neurons from DRG explants. Mean ± SD, *n* = 5. *P < 0.05, ***P < 0.001

It is known that the interdependent relation exists between neurons and target SKM cells. Target SKM cell-derived trophic molecules are particularly important for maintaining normal neuronal function. The neurons themselves have their own survival, development, and metabolism, but the effect of the target SKM on neuronal survival is beyond doubt. In this work, we established a neuromuscular coculture system of organotypic DRG tissue with dissociated SKM cells. This coculture was used to test the neurite outgrowth after exposure of NRG-1β and/or its downstream signaling inhibitors. The neurites were projected radically from the organotypic DRG tissue. Several neurites would be combined together to form a larger neurite bundle. The amount of neurite bundles represents the overall healthy state of the growing DRG explants. After stimulation with NRG-1β and/or its downstream signaling inhibitors, we showed that NRG-1β (20 nmol/L) induced a larger amount of neurite bundles extended from organotypic DRG explants (*P* < 0.001). We also observed that inhibiting each of the three downstream signaling pathways by the distinct inhibitors PD98059, LY294002, or AG490 would block the effects of NRG-1β on neurite bundle growth (*P* < 0.05 or *P* < 0.01). These results also imply that those inhibitors could effectively block the distinct downstream signaling pathways of NRG-1β on neurite outgrowth (Fig. 2-1). The large projections of neurite bundles that sprouted directly from organotypic DRG explants in vitro is an important event and growth pattern of organotypically cultured DRG explants. The promotive effects of NRG-1β on neurite sprouting in this neuromuscular coculture system have significance of NRG-1β on neurite outgrowth in this specific condition.

### Quantification of the migrating neurons from DRG explants

Another indicator, which reflects the overall healthy state of the growing DRG explants, is the amount of migrating neurons from organotypic DRG tissue explants. Hence, we then examined the effects of NRG-1β on neuronal migration from organotypic DRG tissue explants in the established neuromuscular coculture system. We showed that NRG-1β (20 nmol/L) incubation increased the amount of migrating neurons from organotypic DRG tissue explants (*P* < 0.001). We additionally showed that inhibiting each of the three downstream signaling pathways blocked the effects of NRG-1β on neuronal migration (*P* < 0.05 or *P* < 0.001). Therefore, three inhibitors PD98059, LY294002, and AG490 had effective actions on blocking the distinct downstream signaling pathways of NRG-1β on neuronal migration (Fig. 2-2). These results suggested that NRG-1β promoted neuronal migration from organotypic DRG tissue explants in the neuromuscular coculture system, which is different from the in vivo situation of the ontogenesis during nerve-muscle contact. This in vivo process only involved neurites navigating to find their target muscle fibers, rather than neuron cell body migration.

### GAP-43 mRNA and protein expression after NRG-1β incubation

**Fig. 3:**
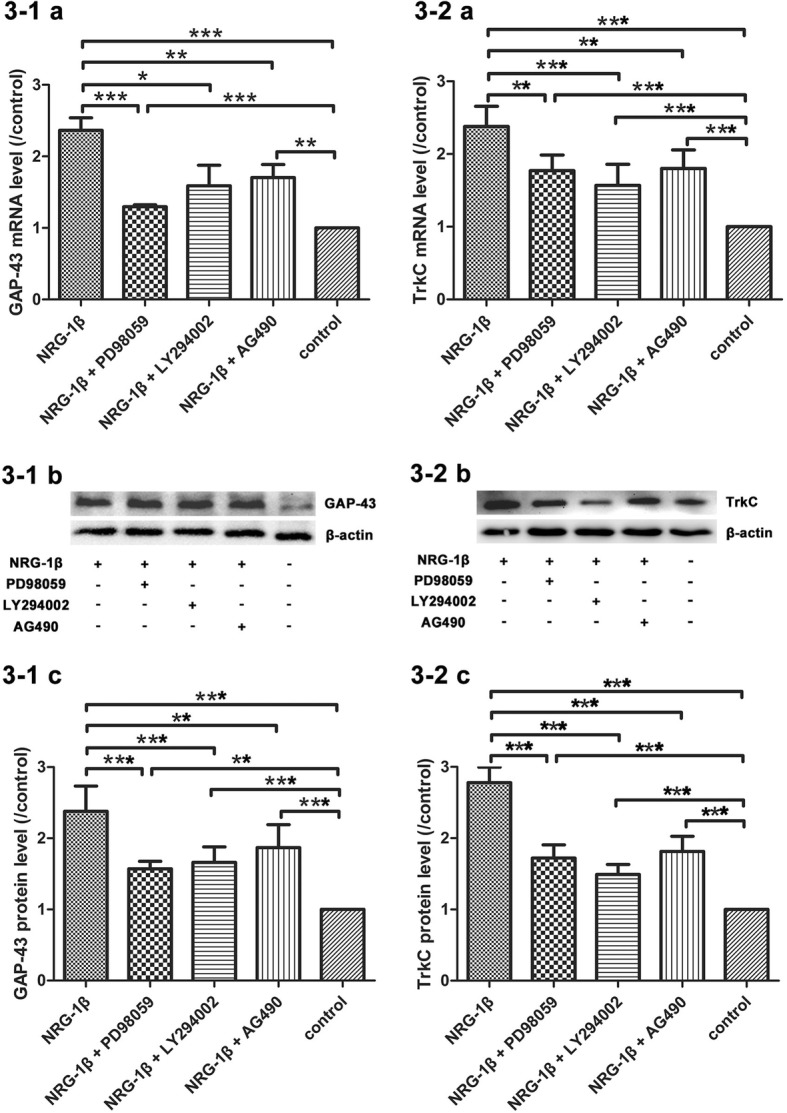
GAP-43 and TrkC mRNA and protein levels. **3-1 a** Quantification of GAP-43 mRNA levels. **3-1 b** Immunoreactive bands of GAP-43 in different experimental conditions. **3-1 c** Quantification of GAP-43 protein levels in different experimental conditions. **3-2 a** Quantification of TrkC mRNA levels. **3-2 b** Immunoreactive bands of TrkC in different experimental conditions. **3-2 c** Quantification of TrkC protein levels in different experimental conditions. Mean ± SD, *n* = 5. *P < 0.05, **P < 0.01, ***P < 0.001

GAP-43, as a kind of neuron-specific calcium-binding protein and actin-binding protein, is highly expressed in the presynaptic membrane. GAP-43 is often used as markers of neuron development, regeneration, and synaptic growth after nerve injury [49, 66]. In this study, we examined the effects of NRG-1β on GAP-43 expression in the neuromuscular coculture system of organotypic DRG tissue with dissociated SKM cells. We showed that NRG-1β (20 nmol/L) incubation elevated both mRNA and protein levels of GAP-43 suggesting the promotive effects of NRG-1β on GAP-43 expression in organotypic DRG tissue explants in this neuromuscular coculture system (*P* < 0.001). We also showed that inhibiting each of the three downstream signaling pathways blocked the effects of NRG-1β on GAP-43 mRNA and protein expression (*P* < 0.05, *P* < 0.01, or *P* < 0.001). The inhibitors PD98059, LY294002, and AG490 could effectively block the distinct downstream signaling pathways of NRG-1β on GAP-43 expression (Fig. 3-1). The elevation of GAP-43 mRNA and protein levels may be the intrinsic mechanism of neurite outgrowth and neuronal migration after NRG-1β administration in the neuromuscular coculture system. Furthermore, the effects of NRG-1β on GAP-43 expression might be through the activation of ERK1/2, PI3K/Akt, and JAK2/STAT3 signaling pathways. To confirm whether these downstream signaling inhibitors hinder neuronal growth state, we investigated the expression of GAP-43 after application of each of the three downstream signaling inhibitors in the absence of NRG-1β. In these cultured conditions, the GAP-43 protein levels were not affected (Additional file 2: Figure S2).

### NRG-1β induced neuromuscular interactions and development of IM fibers

**Fig. 4:**
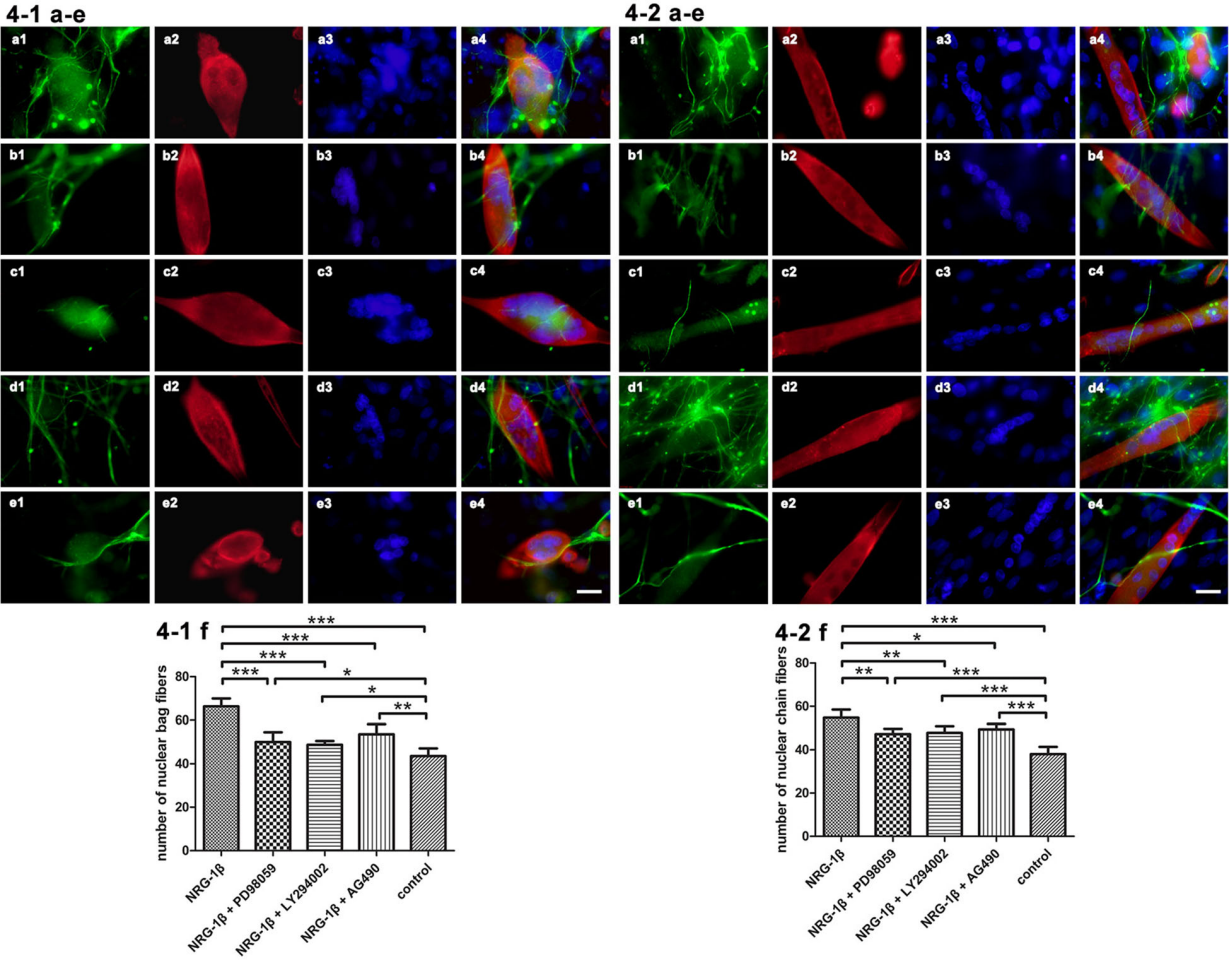
Effects of NRG-1β on intrafusal nuclear bag and chain fiber formation. **a** NRG-1β: **a1** NF-200-IR neurons, **a2** SKM cells, **a3** DAPI, and **a4** overlay of **a1**, **a2**, and **a3**. **b** NRG-1β + PD98059: **b1** NF-200-IR neurons, **b2** SKM cells, **b3** DAPI, and **b4** overlay of **b1**, **b2**, and **b3**. **c** NRG-1β + LY294002: **c1** NF-200-IR neurons, **c2** SKM cells, **c3** DAPI, and **c4** overlay of **c1**, **c2**, and **c3**. **d** NRG-1β + AG490: **d1** NF-200-IR neurons, **d2** SKM cells, **d3** DAPI, and **d4** overlay of **d1**, **d2**, and **d3**. **e** control: **e1** NF-200-IR neurons, **e2** SKM cells, **e3** DAPI, and **e4** overlay of **e1**, **e2**, and **e3**. Scale bar = 20 μm. **4-1 a**–**e** Intrafusal nuclear bag fiber (red) with gathered nuclei (blue) inside and sensory nerve terminals wrapping around the bag fiber surface. **4-2 a**–**e** Intrafusal nuclear chain fiber (red) with linear assembled nuclei (blue) inside and sensory nerve terminals wrapping around the chain fiber surface. **4-1 f** Quantification of number of nuclear bag fibers. **4-2 f** Quantification of number of nuclear chain fibers. Mean ± SD, *n* = 5. *P < 0.05, **P < 0.01, ***P < 0.001

The sensory neurons take distinct paths to their target organs which are critical to the initiation of their axon outgrowth. The intrafusal fiber and the connective tissue capsule surrounding them constitute the muscle spindles, which are specialized sensory receptors and sensitive to muscle length alterations. The IM fibers are of a small diameter and receive sensory innervations as annulospiral endings (from type Ia sensory fiber) and flower-spray endings (from type II sensory fiber), transmitting the movement sense of a moving muscle and position sense of a still muscle, respectively [18]. Here, the differentiation of muscle spindle in the organotypically cultured DRG explants with dissociated SKM cell coculture system was investigated by triple fluorescence staining of NF-200, α-actin, and DAPI. According to the morphology and size of IM fibers in culture in previous study [14] and the growth status of IM fibers in our present coculture system, the two kinds of IM fibers were distinguished as follows: The intrafusal bag fibers are fusiform in shape. The intrafusal chain fibers are thin cylindrical in shape, and the diameter of chain fiber is about 20–30 μm which distinguished from the larger diameter extrafusal fibers. The NRG-1β treatment improved the growth status of DRG neurons which established their contact to their target IM fibers. The results showed that NRG-1β exposure promoted the growth of nerve endings contacting with muscle cells. This in vitro growth pattern morphologically mimicked at the contact area of IM fibers with the sensory nerve terminals in vivo. In this neuromuscular coculture system, we showed that NRG-1β (20 nmol/L) incubation significantly increased the amount of both nuclear bag fibers and nuclear chain fibers (*P* < 0.001). We also showed that inhibiting each of the three downstream signaling pathways would block the effects of NRG-1β on IM fiber formation (*P* < 0.05, *P* < 0.01, or *P* < 0.001). PD98059, LY294002, and AG490 are able to proficiently inhibit the distinct downstream signaling pathways of NRG-1β on IM fiber formation (Fig. 4). To confirm whether these downstream signaling inhibitors hinder the IM fiber formation, we carried out the experiment with only the application of each of the three downstream signaling inhibitors in the absence of NRG-1β. In these culture conditions, the amount of both nuclear bag fibers and nuclear chain fibers was not affected (Additional file 3: Figure S3). Multiple mechanisms and pathways are involved in the process of IM fiber formation. The pathways blocked in this experiment are related to the NRG-1β challenge. In the absence of NRG-1β, inhibition of each of the three downstream signaling pathways did not hinder IM fiber formation.

### The effects of NRG-1β on TrkC mRNA and protein expression

It is known that TrkC expression is usually in large diameter neurons in DRG which is relevant to proprioception. In this study, we examined the effects of NRG-1β on TrkC expression in the neuromuscular coculture system of organotypic DRG tissue with dissociated SKM cells. We showed that NRG-1β (20 nmol/L) incubation increased TrkC mRNA and protein levels (*P* < 0.001). We also showed that inhibiting each of the three downstream signaling pathways would block the effects of NRG-1β on TrkC expression (*P* < 0.01 or *P* < 0.001). The inhibitors PD98059, LY294002, and AG490 are capable to block the distinct downstream signaling pathways of NRG-1β on TrkC mRNA and protein levels (Fig. 3-2). To confirm whether TrkC expression in muscle culture alone or DRG culture alone with NRG-1β stimulation, muscle culture and DRG culture were used separately for these tests. We showed that SKM cells did not express TrkC and DRG expressed TrkC which could be promoted by NRG-1β stimulation (Additional file 4: Figure S4).

### NRG-1β increased TrkC phenotype DRG neurons in neuromuscular cocultures

**Fig. 5:**
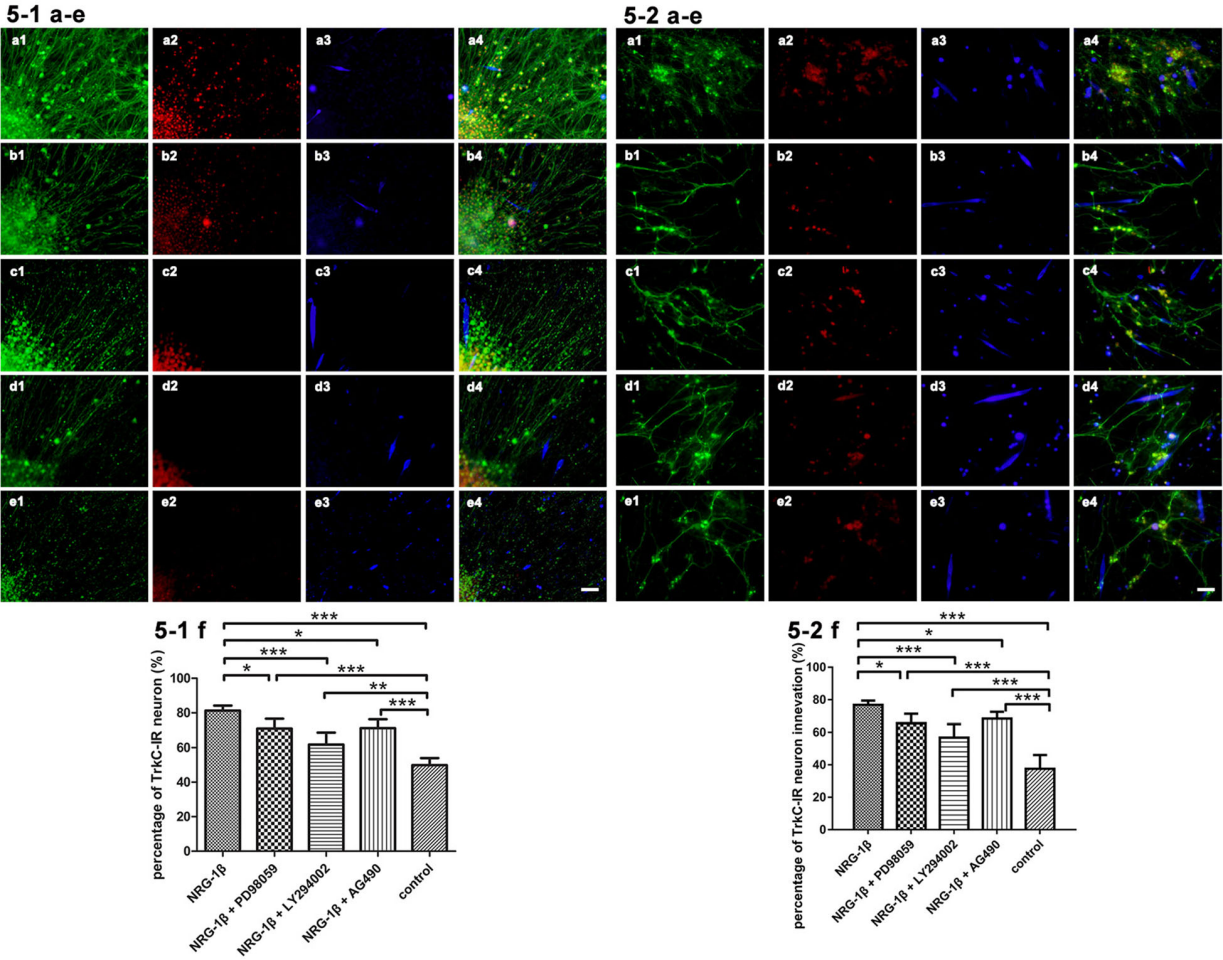
TrkC expression in situ and the percentage of TrkC-IR neuron innervation of muscle fibers. **a** NRG-1β: **a1** NF-200-IR neurons, **a2** TrkC-IR neurons, **a3** SKM cells by α-actin, and **a4** overlay of **a1**, **a2**, and **a3**. **b** NRG-1β + PD98059: **b1** NF-200-IR neurons, **b2** TrkC-IR neurons, **b3** SKM cells by α-actin, and **b4** overlay of **b1**, **b2**, and **b3**. **c** NRG-1β + LY294002: **c1** NF-200-IR neurons, **c2** TrkC-IR neurons, **c3** SKM cells by α-actin, and **c4** overlay of **c1**, **c2**, and **c3**. **d** NRG-1β + AG490: **d1** NF-200-IR neurons, **d2** TrkC-IR neurons, **d3** SKM cells by α-actin, and **d4** overlay of **d1**, **d2**, and **d3**. **e** control: **e1** NF-200-IR neurons, **e2** TrkC-IR neurons, **e3** SKM cells by α-actin, and **e4** overlay of **e1**, **e2**, and **e3**. Scale bar = 50 μm. **5-1 a**–**e** TrkC-IR migrating neurons in different conditions. **5-2 a**–**e** TrkC-IR neurons innervate muscle fibers. **5-1 f** Quantification of the percentage of TrkC-IR migrating neurons. **5-2 f** Quantification of the percentage of TrkC-IR neuron innervation of muscle fibers. Mean ± SD, *n* = 5. *P < 0.05, **P < 0.01, ***P < 0.001

In newborn rats, TrkC-expressing neurons are involved in proprioceptive modulation and can be selectively activated by neurotrophic factors. To test whether TrkC was associated with the development of IM fibers, we analyzed the proportion of the TrkC phenotype DRG neurons in neuromuscular cocultures. At 4 days of coculture age, the in situ expression of TrkC in the neuromuscular coculture was detected by fluorescence labeling of NF-200 and TrkC. We showed that NRG-1β treatment elevated the percentage of TrkC-positive neurons (*P* < 0.001). We also showed that inhibiting each of the three downstream signaling pathways would block the effects of NRG-1β on TrkC in situ expression of DRG neurons in the neuromuscular cocultures of organotypic DRG tissue with dissociated SKM cells (*P* < 0.05 or *P* < 0.001). PD98059, LY294002, and AG490 as the inhibitors of the distinct downstream signaling pathways of NRG-1β could effectively block NRG-1β-induced TrkC expression in situ in DRG neurons (Fig. 5-1).

### NRG-1β increased the number of neurites from TrkC phenotype neurons on muscle fibers

NRG-1β increased TrkC phenotype DRG neurons in the coculture. To further determine whether NRG-1β increased the number of neurites from TrkC phenotype DRG neurons on muscle fibers in neuromuscular cocultures, we analyzed the TrkC-IR (immunoreactive, IR) neurons with their neurites contacted with muscle fibers by triple immunofluorescence staining of NF-200, TrkC, and α-actin. We showed that NRG-1β treatment elevated the percentage of TrkC-positive neurons with their neurites on muscle fibers (*P* < 0.001). We also showed that inhibiting each of the three downstream signaling pathways blocked the effects of NRG-1β on the elevation of the percentage of TrkC-positive neurons with their neurites on muscle fibers (*P* < 0.05 or *P* < 0.001). PD98059, LY294002, and AG490 as the inhibitors of the distinct downstream signaling pathways of NRG-1β could efficiently prevent NRG-1β-induced neurites from TrkC-positive neurons contacted with muscle fibers (Fig. 5-2). Distinct patterns of expression of TrkC are readily apparent in developing DRG from embryonic stage to mediate the biological effects of survival and neurite outgrowth [32]. TrkC is expressed predominantly by large DRG neurons which innervate proprioceptive receptors associated with muscle reflex. These results suggested that NRG-1β incubation induced an increase of anatomical contact between neurites from TrkC-positive neurons and muscle fibers in this coculture system. These phenomena imply that NRG-1β may have specific functions on TrkC-dominated nerve-muscle contact during the process of neurite and muscle fiber outgrowth in vitro. The promotion of TrkC phenotype by NRG-1β might be one of the mechanisms of NRG-1β on the formation of the target IM fibers observed in this study.

## Discussion

Formation of muscle spindles is a multistep process involved in coordinated forth-and-back communication between developing sensory nerve endings and target IM fibers. The development of IM fibers is a key step in the formation of the muscle spindles and then finally for the construction of the stretch reflex arc. The development and formation of IM fibers must rely on the exact nerve-muscle contact and sufficient trophic factors. NRG-1 signaling as an anterograde differentiation and growth factor is a key regulator on IM fiber development [9], muscle spindle formation [10], and muscle spindle maturation [6]. IM fibers associated with their proprioceptive sensory neurons compose the sensory circuit of the neuromuscular reflex arc [20]. A wide range of diseases causing this sensory circuit portion damage will result in motor dysfunction [24]. However, reconstruction of the damaged sensory circuit is not defined now. In this study, we established a coculture system, using organotypic DRG tissue with dissociated SKM cells in the same culture well, to imitate development of sensory neuromuscular contact during the ontogenesis. The results showed that NRG-1β promoted neurite outgrowth and neuronal migration from the organotypic DRG explants and that this correlated with an induction of GAP-43 expression. The modulating effects of NRG-1β on TrkC DRG neuronal phenotype may link to promote IM fiber (nuclear bag fiber and nuclear chain fiber) formation. In addition, the effects of NRG-1β on IM fiber formation are correlated to the activation of ERK1/2, PI3K/Akt, and JAK2/STAT3 intracellular signaling pathways. These effects were tested by using ERK1/2 inhibitor PD98059, PI3K inhibitor LY294002, and JAK2 inhibitor AG490, respectively. These inhibitors could effectively block the distinct downstream signaling of NRG-1β.

NRG-1 plays a critical role in controlling migration of neuronal progenitors and neurons during development [67]. The migration of neurons to their final destinations is essential for maintenance of the morphogenesis of the nervous system during development. This navigating path is complex which depends on the cellular environment [68]. During embryogenesis, projecting neurites to their target organs could be interfered by many factors [69]. In order to maintain the normal function of the nerve-target tissue system, modification of neuron migration and the neurite projecting environment that helps neurite in projecting or efficient pathfinding to its target is essential. NRG-1β, because of its neurite outgrowth promotive actions, might be one of the candidates for helping neurites from DRG neurons to their target tissues, as well as promoting distinct Trk neuron migration to their target positions. Several studies have shown that NRG-1 has specific and important actions on formation, maturation, function, and maintenance of IM fibers or muscle spindles [6, 9, 10, 13, 14, 60], which provide us relevant references for designing and completing this experiment of IM fiber formation in the present of sensory neurons in vitro. The similar actions of NRG-1β on formation of nuclear bag fibers and nuclear chain fibers were confirmed in our established neuromuscular coculture. The results of our present study provided basic and crucial experimental evidence for studying classification on IM fibers, in vitro innervation patterns, and neuromuscular interactions with selective sensory innervation. In the present study, neuron migration as well as neurite projecting under the promotion of NRG-1β is sufficient to guide to innervate its exact target tissue to restore neuromuscular contact. Likewise, elevated GAP-43 levels in DRG neurons resulted in the nerve-muscle contact as well as the promotion of neurite projection and neuron migration. Furthermore, it is tempting to speculate that these neuronal behaviors and molecular events might be involved in the formation of IM fibers.

The alterations of a muscle length could be detected by the specialized proprioceptor intrafusal fibers. They are innervated by sensory axons from DRG neurons. The biological condition of the IM fibers is one of the vital elements of full functional recovery from injury. IM fiber formation depends on the growth environments. The existence of sensory innervation and the addition of neurotrophic factors are dependent on environmental factors for the formation of IM fibers during development. NRG-1β, released from sensory nerve terminals, is necessary for muscle spindle formation by activating ErbB receptors in muscle cells [60]. These observations suggested that neurogenic NRG has multifunctional actions on maintaining the specific functions of target IM fibers. To investigate the specific effects of NRG-1β on the formation of IM fibers in the coculture, triple fluorescence staining of NF-200, α-actin, and DAPI was used to determine the distribution of nucleus and the sensory nerve terminal. After NRG-1β exposure, the formation of both two types of IM fiber was observed. The DRG nerve terminals not only grow longer and sprout more branches to innervate the muscle cell with NRG-1β treatment, but also form more annulospiral endings and flower-spray endings around the muscle fibers. These nerve endings were observed in mouse soleus muscle spindle in animal models in previous study [22]. The endings of these nerve fibers contact the nucleus region of the IM fibers, where the muscle fibers only stretch to generate information of the position of one’s muscle, but do not contract to generate skeletal movement. These results show that in addition to the effect of promoting the neurite outgrowth, NRG-1β promoted the formation of IM fibers with the sensory DRG neurons in neuromuscular cocultures. While the migrating neurons could target the muscle cells by the pathfinding route, the formation of IM fibers had more significance in these neuromuscular cocultures with DRG neurons, for the IM fibers are actually innervated by sensory neurons in vivo. Our results do not rule out that the nuclear bag and nuclear chain fibers could only be observed when the SKM is cocultured with sensory neurons, but they are consistent with the hypothesis that the formation of IM fibers critically depends on the growth environments, at least in part, for the existence of specific proprioceptive neurons and the trophic factor NRG-1β.

It has been shown that activation of receptor could induce axon sprouting to re-innervate SKM [70] as well as promote sensory and motor axonal regeneration after peripheral injury [71]. When particular signaling by neurotrophic factors was lost, mice muscle spindles fail to develop due to lack of sensory neurons [60]. Our results have shown that the exogenous NRG-1β promoted TrkC expression and increased the number of TrkC-positive neurons innervating target muscle cells. While the IM fiber formation is required for neuronal migration and neurite projection to the target muscle cells, TrkC might be a particular receptor that responds to neurotrophic factors and induces the formation of intrafusal fibers. As our analysis of TrkC DRG neuronal phenotype participates in the development and formation of intrafusal fibers, there might be a role for SKM cells on promoting the expression of TrkC in DRG neurons. TrkC expression in DRG neurons promoted by the target SKM cells might also play a significant role, suggesting a mutual exchange of nerve-muscle molecular signals during development.

Another interesting phenomenon is that the migrating neurons directly form DRG explants scattered around the DRG explants in the peripheral area and are distributed between the growing muscle fibers. In fact, neuronal cell bodies do not migrate in vivo for the neuron to form a synapse. During this process, only the axons migrate along with a definite path to find their target to form synapses or other specific junctions. However, neuronal cell bodies can migrate from the organotypic DRG explants in vitro in this study. This is a special coculture system which provides migration environment that let neuronal cell bodies migrate from DRG explants to the peripheral area, which is different from the conditions in vivo. In this neuromuscular coculture system, neuronal migration has specific significance for the neuromuscular interaction, because neuronal cell bodies can directly send axons to the adjacent muscle fibers, which made the higher innervation efficiency in this specific coculture condition. The amount of migrating neuronal cell bodies represents the growth state of the DRG explants in our present study.

## Conclusion

In conclusion, NRG-1β promotes outgrowth of neurites and migration of neurons from the organotypic DRG explants, and this correlated with an induction of GAP-43 expression. Based on the promotion of neuronal outgrowth, NRG-1β induces IM fiber formation by modulating TrkC DRG neuronal phenotype in neuromuscular cocultures. The effects produced by NRG-1β in this neuromuscular coculture system provide new data for the therapeutic potential on IM fiber formation after muscle injury.

## Additional files

Additional file 1: **Figure S1.** An example of images from NRG-1β treated sample to show how to count the percentage of TrkC-positive neurons. a MAP2 fluorescence labeling for all the migrating neurons (green). b TrkC fluorescence labeling (red). c Overlay of a and b (orange color shows TrkC-positive neurons). (TIF 520 kb)

Additional file 2: **Figure S2.** Western blot assay for GAP-43 protein levels in the absence of NRG-1β. a Immunoreactive bands for GAP-43. b Quantification of GAP-43 protein levels. Mean ± SD, *n* = 5. (TIF 177 kb)

Additional file 3: **Figure S3.** Quantification of number of nuclear bag and chain fibers in the absence of NRG-1β. a Number of nuclear bag fibers in the absence of NRG-1β. b Number of nuclear chain fibers in the absence of NRG-1β. Mean ± SD, n = 5. (TIF 224 kb)

Additional file 4: **Figure S4.** Western blot assay for TrkC protein levels in SKM culture alone or DRG culture alone in the presence or absence of NRG-1β. a Immunoreactive bands for TrkC. b Quantification of TrkC protein levels. Mean ± SD, n = 5. *P < 0.001. (TIF 164 kb)

## Abbreviations

DAPI: 4′,6-Diamidino-2-phenylindole
DRG: Dorsal root ganglion
DRGs: Dorsal root ganglia
E15: Embryonic day 15
ERK1/2: Extracellular signal-regulated protein kinase 1/2
GAP-43: Growth-associated protein 43
GAPDH: Glyceraldehyde-3-phosphate dehydrogenase
JAK2: Janus kinase 2
MAP2: Microtubule-associated protein 2
NMJ: Neuromuscular junction
NRG-1: Neuregulin-1
PBS: Phosphate buffer saline
PI3K: Phosphatidylinositol 3-kinase
SKM: Skeletal muscle
STAT3: Signal transducer and activator of transcription 3
TRITC: Tetramethyl rhodamin isothiocyanate
Trk: Tropomyosin-related kinase
TrkC: Tyrosine kinase receptor C
