# Supplementary figures and images for: The effects of neuregulin-1β on intrafusal muscle fiber formation in neuromuscular coculture of dorsal root ganglion explants and skeletal muscle cells
Source: Skelet Muscle. 2018 Sep 15;8:29. doi: 10.1186/s13395-018-0175-9 (PMC6139134; doi:10.1186/s13395-018-0175-9)

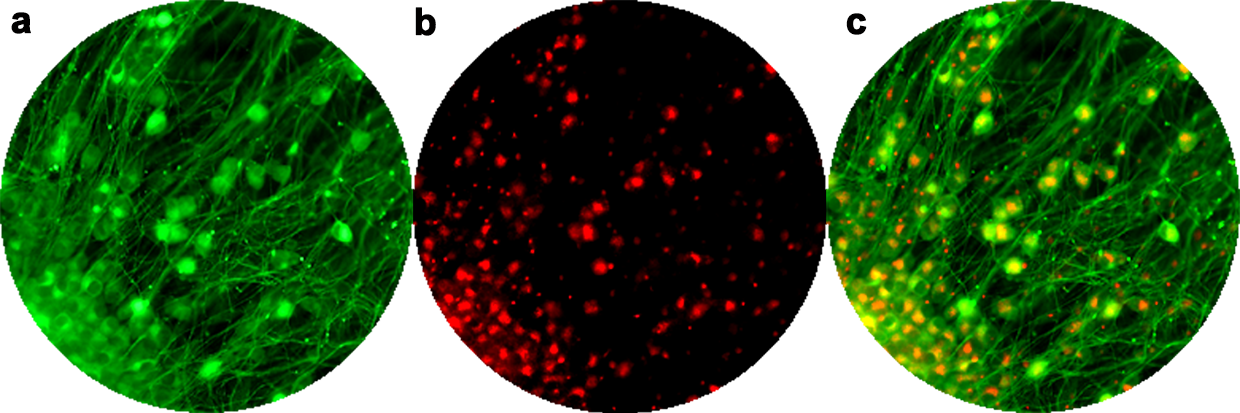

Supplement: Supplementary file 1 — Figure S1. An example of images from NRG-1β treated sample to show how to count the percentage of TrkC-positive neurons. a MAP2 fluorescence labeling for all the migrating neurons (green). b TrkC fluorescence labeling (red). c Overlay of a and b (orange color shows TrkC-positive neurons). (TIF 520 kb) [file 13395_2018_175_MOESM1_ESM.tif]

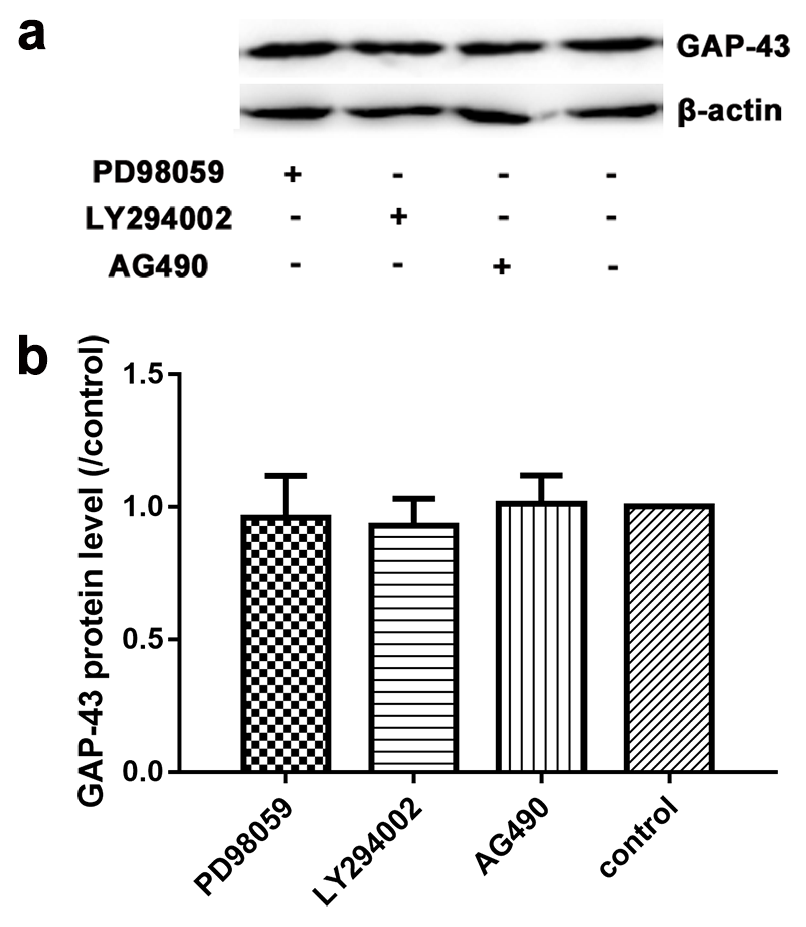

Supplement: Supplementary file 2 — Figure S2. Western blot assay for GAP-43 protein levels in the absence of NRG-1β. a Immunoreactive bands for GAP-43. b Quantification of GAP-43 protein levels. Mean ± SD, n = 5. (TIF 177 kb) [file 13395_2018_175_MOESM2_ESM.tif]

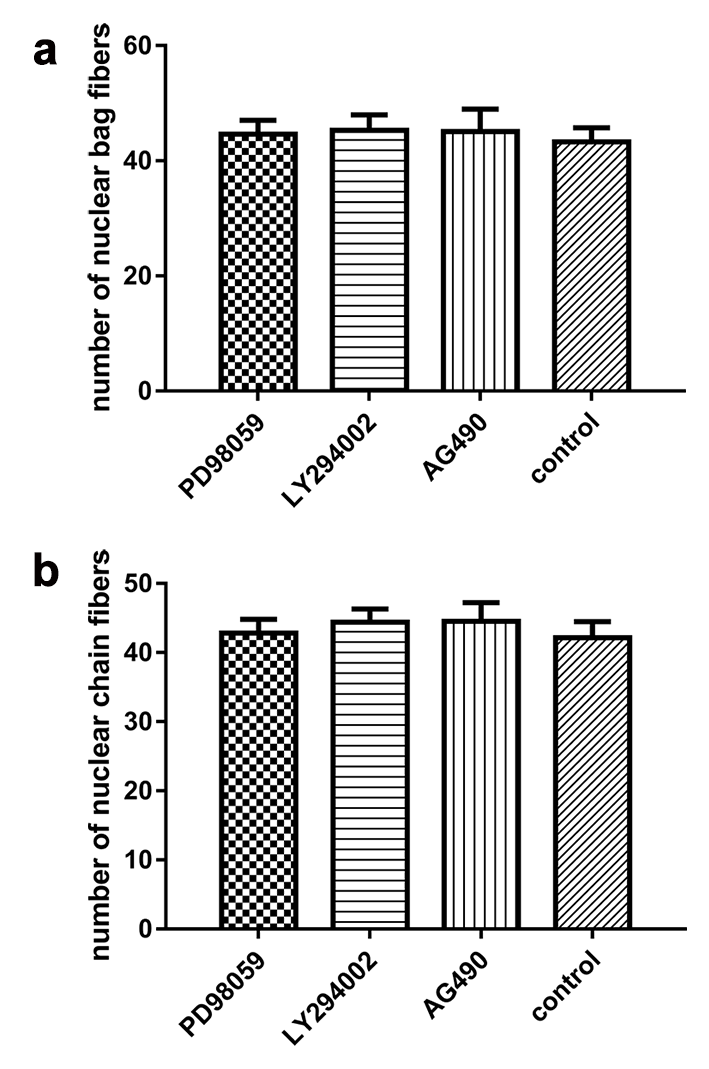

Supplement: Supplementary file 3 — Figure S3. Quantification of number of nuclear bag and chain fibers in the absence of NRG-1β. a Number of nuclear bag fibers in the absence of NRG-1β. b Number of nuclear chain fibers in the absence of NRG-1β. Mean ± SD, n = 5. (TIF 224 kb) [file 13395_2018_175_MOESM3_ESM.tif]

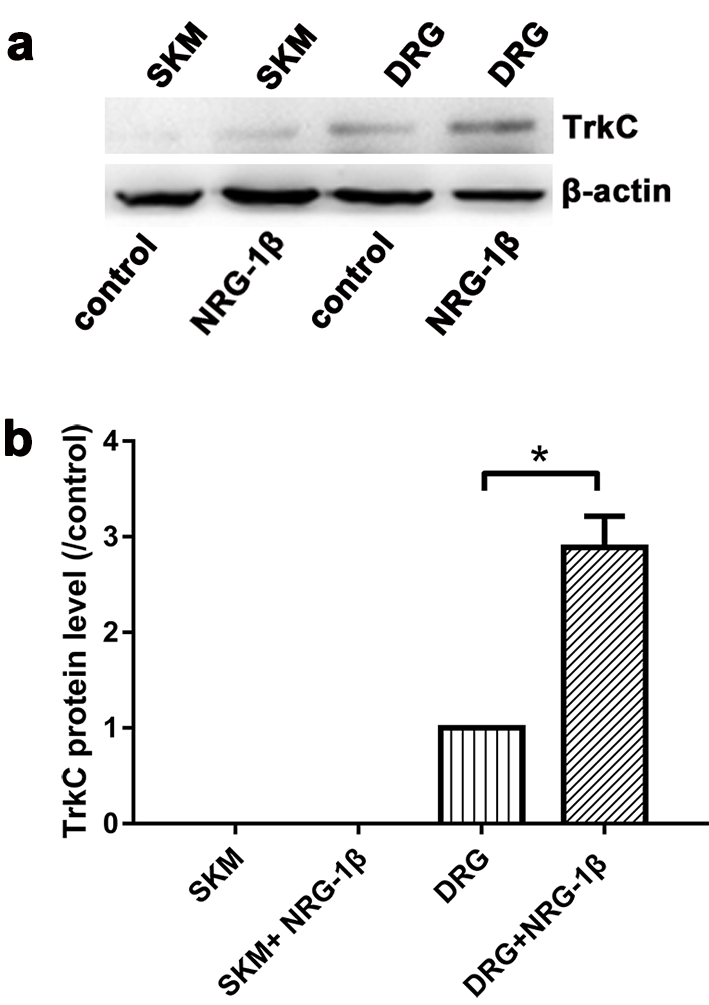

Supplement: Supplementary file 4 — Figure S4. Western blot assay for TrkC protein levels in SKM culture alone or DRG culture alone in the presence or absence of NRG-1β. a Immunoreactive bands for TrkC. b Quantification of TrkC protein levels. Mean ± SD, n = 5. *P < 0.001. (TIF 164 kb) [file 13395_2018_175_MOESM4_ESM.tif]
